# Right ventricular strain analysis in acute pulmonary embolism and its role for late complications

**DOI:** 10.1093/ehjimp/qyag166

**Published:** 2026-09-22

**Authors:** Ioannis T Farmakis, Lina-Marie Brandes, Karsten Keller, Gerald S Laux-Winter, Sara Bombace, Philipp Lurz, Stavros V Konstantinides, Lukas Hobohm

**Affiliations:** Center for Thrombosis and Hemostasis (CTH), University Medical Center of the Johannes Gutenberg University, 55131 Mainz, Germany; Department of Medicine I, LMU University Hospital, LMU Munich, Germany; Department of Cardiology, University Medical Center of the Johannes Gutenberg-University Mainz, 55131 Mainz, Germany; Department of Cardiology, University Medical Center of the Johannes Gutenberg-University Mainz, 55131 Mainz, Germany; Center for Thrombosis and Hemostasis (CTH), University Medical Center of the Johannes Gutenberg University, 55131 Mainz, Germany; Department of Cardiology, University Medical Center of the Johannes Gutenberg-University Mainz, 55131 Mainz, Germany; Department of Cardiology, University Medical Center of the Johannes Gutenberg-University Mainz, 55131 Mainz, Germany; Department of Cardiology, University Medical Center of the Johannes Gutenberg-University Mainz, 55131 Mainz, Germany; Department of Cardiology, University Medical Center of the Johannes Gutenberg-University Mainz, 55131 Mainz, Germany; Center for Thrombosis and Hemostasis (CTH), University Medical Center of the Johannes Gutenberg University, 55131 Mainz, Germany; Department of Cardiology, Democritus University of Thrace, Alexandroupolis, Greece; Center for Thrombosis and Hemostasis (CTH), University Medical Center of the Johannes Gutenberg University, 55131 Mainz, Germany; Department of Cardiology, University Medical Center of the Johannes Gutenberg-University Mainz, 55131 Mainz, Germany

**Keywords:** pulmonary embolism, post-PE impairment, CTEPH, RV systolic strain, ventriculoarterial coupling

## Abstract

**Aims:**

Acute pulmonary embolism (PE) can result in long-term complications, including post-PE impairment (PPEI) and chronic thromboembolic pulmonary hypertension (CTEPH). Right ventricular global longitudinal strain (RVGLS) is a sensitive measure of RV myocardial deformation that may detect subclinical dysfunction and predict adverse outcomes; however, its value in the follow-up of post-PE assessment has not been yet elucidated.

**Methods and results:**

In this prospective sub-study of the FOCUS cohort, 175 consecutive PE survivors from the Mainz centre underwent echocardiographic follow-up at 3 and 12 months, including speckle-tracking assessment of RVGLS, RV free wall strain (RVFWLS), and strain-derived ventriculoarterial coupling indices. Conventional RV parameters, volumetric measures, and clinical outcomes were recorded. Overall, 175 patients were included, of whom 16 developed PPEI or CTEPH during follow-up. RV function improved over time in patients without PPEI/CTEPH, including increases in RVGLS (from −16 to −18%), RVFWLS, ejection fraction, and strain-based ventriculoarterial coupling, accompanied by reductions in RV volumes. In contrast, patients who developed PPEI/CTEPH (*n* = 16) displayed progressive deterioration of RV strain and strain-derived coupling despite relatively preserved TAPSE-based indices. At 3 months, RVGLS was significantly associated with the combined PPEI/CTEPH endpoint over 2 years (OR 1.10, 95% CI 1.01–1.21), whereas strain-derived ventriculoarterial coupling parameters demonstrated borderline associations [RVGLS/systolic pulmonary artery pressure (sPAP): OR 0.11, 95% CI 0.01–0.91; RVFWLS/sPAP: OR 0.16, 95% CI 0.02–1.17]. TAPSE/sPAP was not associated with the outcome.

**Conclusion:**

RV strain and strain-derived ventriculoarterial coupling may provide sensitive early markers of maladaptive RV remodelling after acute PE and were associated with the development of PPEI/CTEPH, highlighting their potential value in post-PE follow-up strategies.

## Graphical Abstract

**Graphical Abstract:**
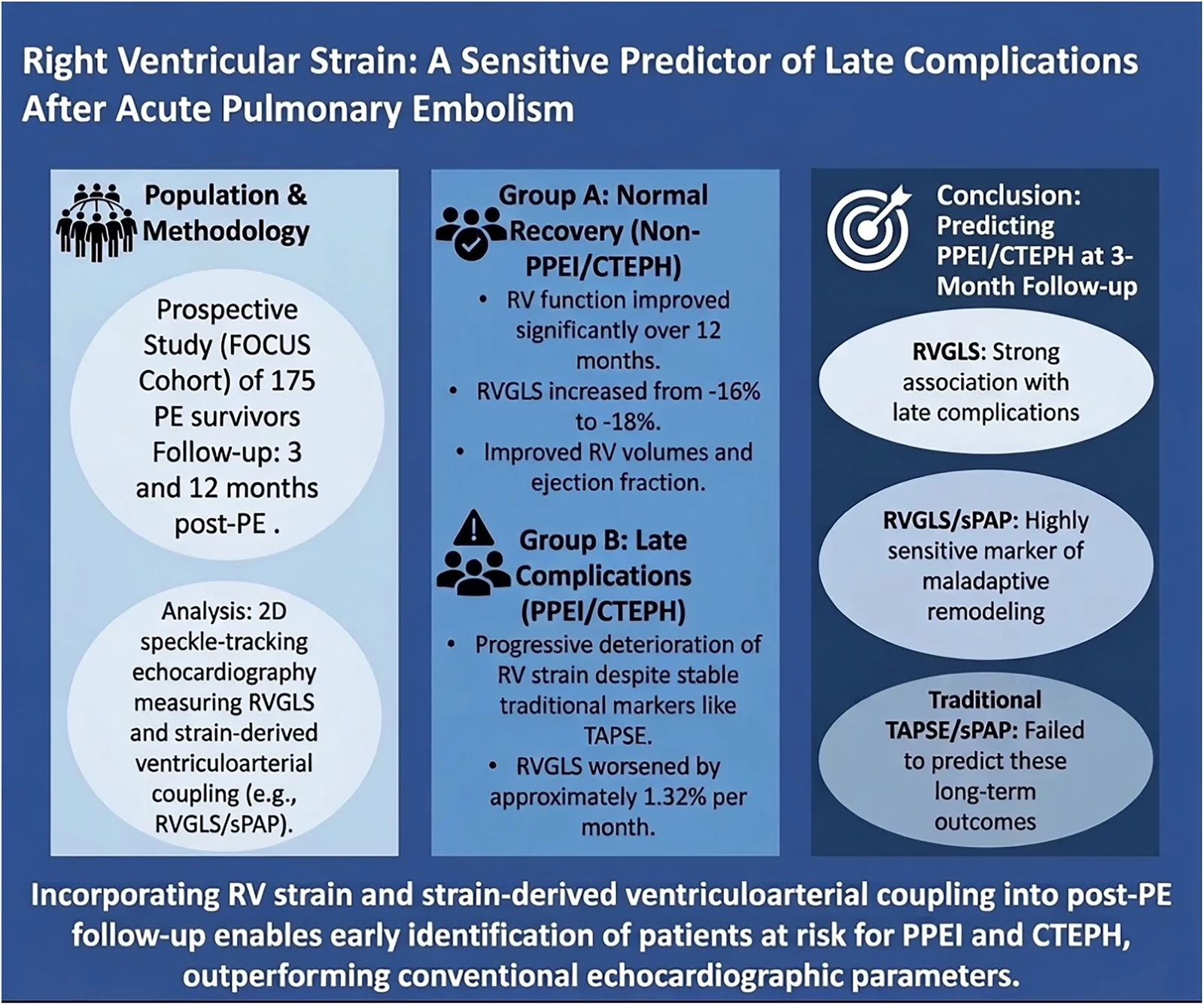

## Introduction

Acute pulmonary embolism (PE) remains a significant cause of morbidity and mortality worldwide,^1^ necessitating precise risk stratification to guide therapeutic decisions.^2,3^ While established prognostic markers like haemodynamic instability and right ventricular (RV) dysfunction are integral to predicting short-term mortality, there is a growing need to identify markers that predict long-term complications, such as post-PE impairment (PPEI) and chronic thromboembolic pulmonary hypertension (CTEPH).^4,5^ These conditions significantly impact quality of life, functional capacity, and long-term outcomes in survivors of PE.^6–8^

RV longitudinal strain (RVLS) is an innovative echocardiographic parameter that quantifies RV regional myocardial deformation. Compared with traditional measures of RV function, RVLS offers a sensitive and reproducible assessment of RV performance and is angle-independent. Although RVLS is influenced by loading conditions, it may detect subtle changes in RV myocardial function making it a reliable tool for evaluating RV performance that offers significant prognostic value in various cardiovascular conditions, including PE.^9–11^

Following PE, RV dysfunction plays a pivotal role in determining both short-term mortality and long-term complications such as PPEI and CTEPH.^12^ RV global longitudinal strain (RVGLS) may provide an enhanced understanding of these outcomes by offering a detailed assessment of RV mechanics, surpassing the limitations of conventional RV function parameters like tricuspid annular plane systolic excursion (TAPSE).^13^

The primary objective of this study was to describe the trajectory of RVGLS during short- and long-term follow-up after PE and to investigate its association with the development of PPEI and CTEPH. Secondary analyses assessed the relationship of RV strain parameters with baseline PE severity, quality of life, rehospitalization, and all-cause mortality.

## Material and methods

### Patient cohort and study design

The FOCUS study (German Clinical Trials Registry: DRKS00005939) prospectively enrolled consecutive, unselected patients with confirmed acute symptomatic PE.^12^ For this *post hoc* sub-study, echocardiography images were used from patients included between September 2014 and October 2018 in the centre of Mainz only. Participants were followed over a 2-year period according to a predefined schedule, which included clinical, functional, laboratory, and echocardiographic assessments, alongside evaluations of health status at five time points: enrolment, hospital discharge, and at 3, 12, and 24 months. For this sub-study, RV strain analyses were performed on echocardiographic images obtained at three of the five time points of the parent study (enrolment, 3 months, and 12 months). The study protocol did not impose specific diagnostic or therapeutic interventions; instead, patients receive care according to the standard clinical practices of the participating centres, following current guideline recommendations. Patients with asymptomatic PE or a prior diagnosis of CTEPH were excluded from participation.

### Echocardiographic assessment

Standard echocardiographic parameters such as right atrial (RA) end-systolic area, RV end-diastolic diameter, thickness of RV free wall, RV/left ventricular (LV) end-diastolic ratio, TAPSE, tricuspid regurgitation velocity, and systolic pulmonary artery pressure (sPAP) estimation were assessed in accordance with ESC guidelines. To evaluate RV strain, independent investigators (I.T.F., L.-M.B., K.K., G.S.L., S.B., and L.H.) performed two-dimensional speckle-tracking echocardiography using the Philips QLAB 15.0 offline software programme (Philips Healthcare Medical Imaging System Andover, MA, USA). The region of interest for the RV was set semi-automatically in the apical four-chamber view and manually adjusted if necessary. The RV global longitudinal strain (RVGLS), the RV free wall longitudinal strain (RVFWLS), the RV septal wall longitudinal strain (RVSWLS), and the strain of the six individual segments (basal, mid, and apical segments of the septum and the free lateral wall) were measured (*Figure 1*). In addition, we also measured the RV end-diastolic volume, RV end-systolic volume, and RV ejection fraction. Using the same methodology as for the RV, the LV global longitudinal strain (LVGLS) was also assessed.

**Figure 1:**
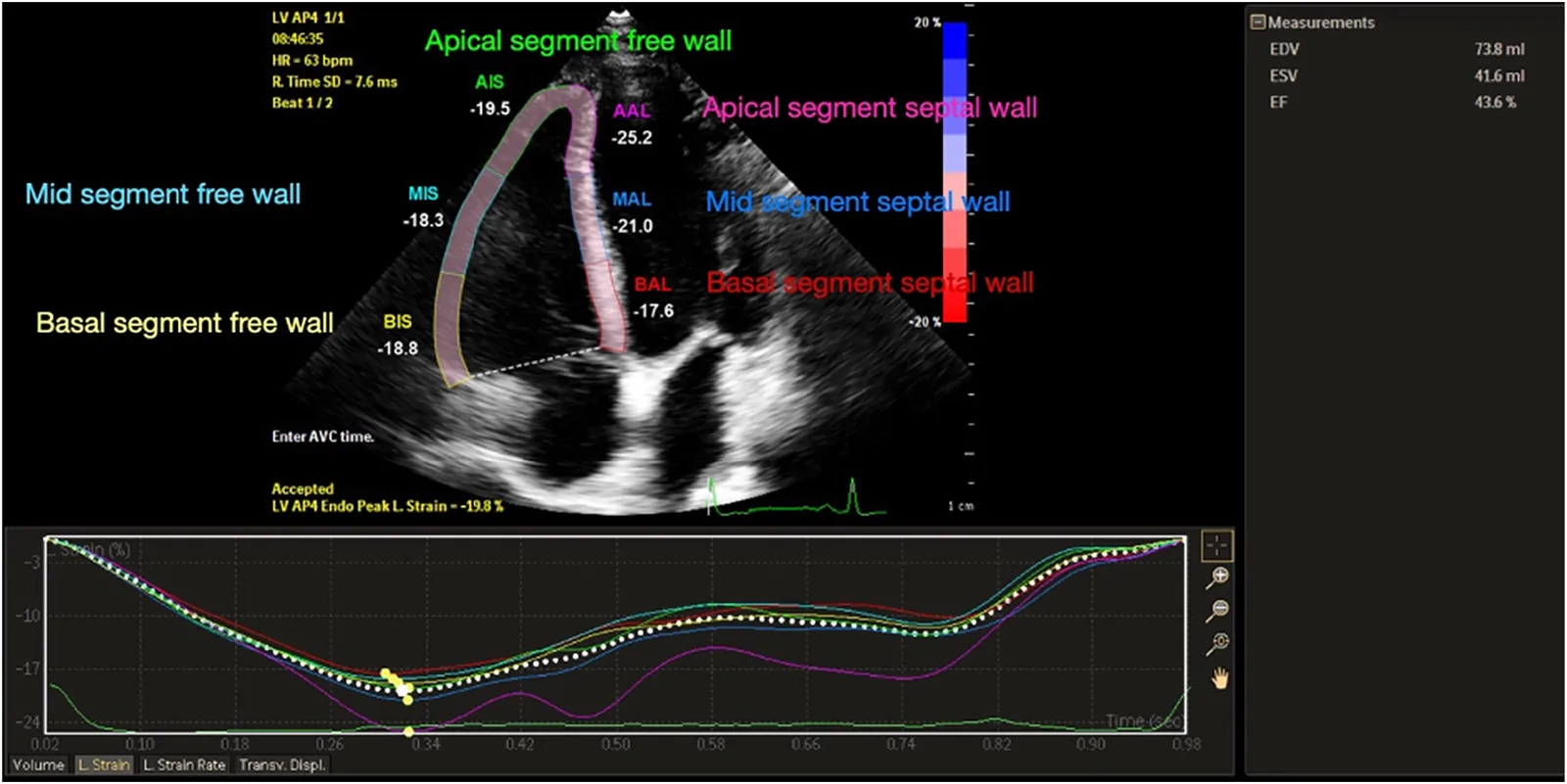
RV segmentation in the offline echocardiographic strain analysis.

Additionally, strain rate, which represents myocardial deformation or contraction per second, and the time to peak longitudinal systolic strain (time to PLSS) were measured. The latter describes the time from the beginning of the QRS complex to the peak strain value within the cardiac cycle. From the standard deviations of the times to the PLSS of the six segments, the RV peak systolic strain dyssynchrony index can be calculated. This index serves as a measure of mechanical dispersion and provides information about the synchrony of ventricular contraction. RVGLS to sPAP ratio, RVFWLS to sPAP ratio, and TAPSE to sPAP ratio were calculated as surrogate markers for ventriculoarterial coupling.^14–16^

RVGLS and RVFWLS were considered the main strain parameters, whereas additional strain-derived indices were analysed exploratorily to assess regional and temporal aspects of RV mechanical adaptation following acute PE.

### Outcomes

The diagnosis of CTEPH during the 2-year follow-up period was confirmed by an independent adjudication committee, applying a predefined adjudication charter and adhering to contemporaneous guideline recommendations in place at the time of the study.^17^ PPEI was defined by either persistence or worsening to the most severe category of disease, requiring the presence of both (i) at least one echocardiographic marker of RV or pulmonary circulatory involvement and (ii) at least one clinical, functional, or laboratory abnormality documented at two successive follow-up assessments. Importantly, the echocardiographic criteria for the definition of PPEI comprised of RV basal diameter, RA end-systolic area, TAPSE, tricuspid regurgitation jet velocity, LV eccentricity index, estimated RA pressure, and presence of pericardial effusion and did not involve any of the aforementioned strain or coupling parameters.

Disease-specific quality of life was assessed using the PEmb-QoL score.^18^ All-cause hospitalization during the course of follow-up was also registered among participants.

### Statistical analysis

Frequencies and percentages were used to summarize categorical variables, while continuous variables were described as means with standard deviation or medians with interquartile range (first and third quartile), depending on their distribution. Unadjusted between-group comparisons (patients diagnosed with PPEI/CTEPH during follow-up vs. no PPEI/CTEPH) were analysed through the Fisher’s exact test, Pearson’s χ^2^ test, and Wilcoxon rank sum test, as appropriate. The Spearman’s rank correlation coefficient and linear regression were used to describe association between RVGLS and markers of worse prognosis at baseline [troponin, brain natriuretic peptide (BNP), and simplified PE severity index (sPESI)]. To study the effect of follow-up time on the evolution of echocardiographic parameters, we used linear mixed-effects models with the follow-up visit as a fixed effect and a patient-specific random intercept to account for the associations of repeated measurements in the same patient. Analyses were stratified according to the PPEI/CTEPH status. Lastly, linear and logistic regression analyses were performed to study the effect of RV strain echocardiography parameters on the outcomes after multivariable adjustment for age and sex. We included all the available observations without imputation under the assumption that data were missing at random. A two-tailed *P*-value of <0.05 was considered to set the significance level. All analyses were performed using R (version 4.4.2).

## Results

### Baseline characteristics

The FOCUS study cohort included 272 patients enrolled in Mainz. In total, 97 (35.7%) patients were excluded because echocardiographic images were unavailable or if strain analyses could not be performed due to poor image quality (baseline differences between included and excluded patients are reported in Supplementary data online, *Table S1*). After applying the exclusion criteria, 175 patients remained with a baseline visit, and 144 had both a baseline visit and at least one follow-up assessment at 3 or 12 months (65 patients had both 3-month and 12-month follow-up available, 53 patients had only 3-month follow-up available, and 26 patients had only 12-month follow-up available). The median age was 61 years with slight majority of male patients (59%). A history of venous thromboembolism was observed in 32%, while 13 and 10% suffered from chronic pulmonary or chronic heart disease, respectively. RV dysfunction on imaging was reported in 99 patients (57%), and 81 patients (49%) had elevated troponin levels. Baseline characteristics of the patients are listed in *Table 1*.

**Table 1:** Baseline characteristics of patients

Characteristics	*N* = 175^a^
Age	61 (51, 73)
Women	71/175 (41%)
BMI (kg/m^2^)	28 (26, 33)
Systolic blood pressure (mmHg)	138 (124, 154)
Diastolic blood pressure (mmHg)	81 (74, 91)
SpO_2_ at room air (%)	96.0 (93.0, 97.0)
Either troponin T or troponin I elevation	81/167 (49%)
Right ventricular dysfunction on imaging^b^	99/175 (57%)
sPESI	
0	97/175 (55%)
1–2	73/175 (42%)
3–4	5/175 (3%)
PE early mortality risk	
Low	41/175 (23%)
Intermediate	129/175 (74%)
High	5/175 (2.9%)
Cancer	12/175 (6.9%)
Oestrogen-containing medication	17/175 (9.7%)
History of pregnancy or lactation	2/175 (1.1%)
History of long-distance travel	21/175 (12%)
Major trauma or major surgery (last 30 days)	28/175 (16%)
History of immobilization	54/175 (31%)
History of VTE	56/175 (32%)
Chronic pulmonary disease	22/175 (13%)
History of CHF or CHD	18/174 (10%)
Arterial hypertension	109/175 (62%)
Diabetes mellitus	30/175 (17%)
Chronic liver disease	9/175 (5.1%)
History of CKD or eGFR <50 at baseline	32/175 (18%)
Chronic inflammatory disease	19/175 (11%)
Smoker	32/175 (18%)

^a^Median (IQR); *n*/*N* (%).

^b^Signs of RV dysfunction on computed tomography pulmonary angiography or echocardiography were diagnosed at the participating sites based on local protocols and in adherence with European and national guidelines valid at the time of patient enrolment.

BMI, body mass index; CHD, coronary heart disease; CHF, congestive heart failure; CKD, chronic kidney disease; eGFR, estimated glomerular filtration rate; PE, pulmonary embolism; sPESI, simplified pulmonary embolism severity index; SpO2, peripheral oxygen saturation measured by pulse oximetry; VTE, venous thromboembolism.

### Echocardiographic parameters at baseline and follow-up and association with risk of the index event

Echocardiographic characteristics at baseline and follow-up are provided in *Table 2* and Supplementary data online, *Table S2*. Regarding strain parameters of all patients, the RVGLS was −16%, the RVFWLS was −16%, and the RVSWLS was −18% at the time of the acute PE. The median LVGLS was −17.2%. Patients with a high early mortality risk exhibited significantly worse RV strain values compared with those at low risk, with a stepwise deterioration across risk categories for RVGLS [low risk: −20% (−24 to −16), intermediate risk: −15% (−19 to −11), high risk: −12% (−12 to −10)], RVFWLS [low risk: −20% (−25 to −16), intermediate risk: −15% (−20 to −10), high risk: −10% (−13 to −7)], and RVSWLS [low risk: −20.3% (−24.8 to −17.6), intermediate risk: −16.3% (−21.0 to −12.6), high risk: −14.5% (−14.9 to −14.4)] (*Figure 2*). RVGLS showed a moderate to strong correlation with biomarkers of worse prognosis in the acute phase including troponin levels (*ρ* = 0.48, *P* < 0.001) and BNP (*ρ* = 0.49, *P* < 0.001), and a weak correlation with the sPESI score (*ρ* = 0.21, *P* = 0.006).

**Figure 2:**
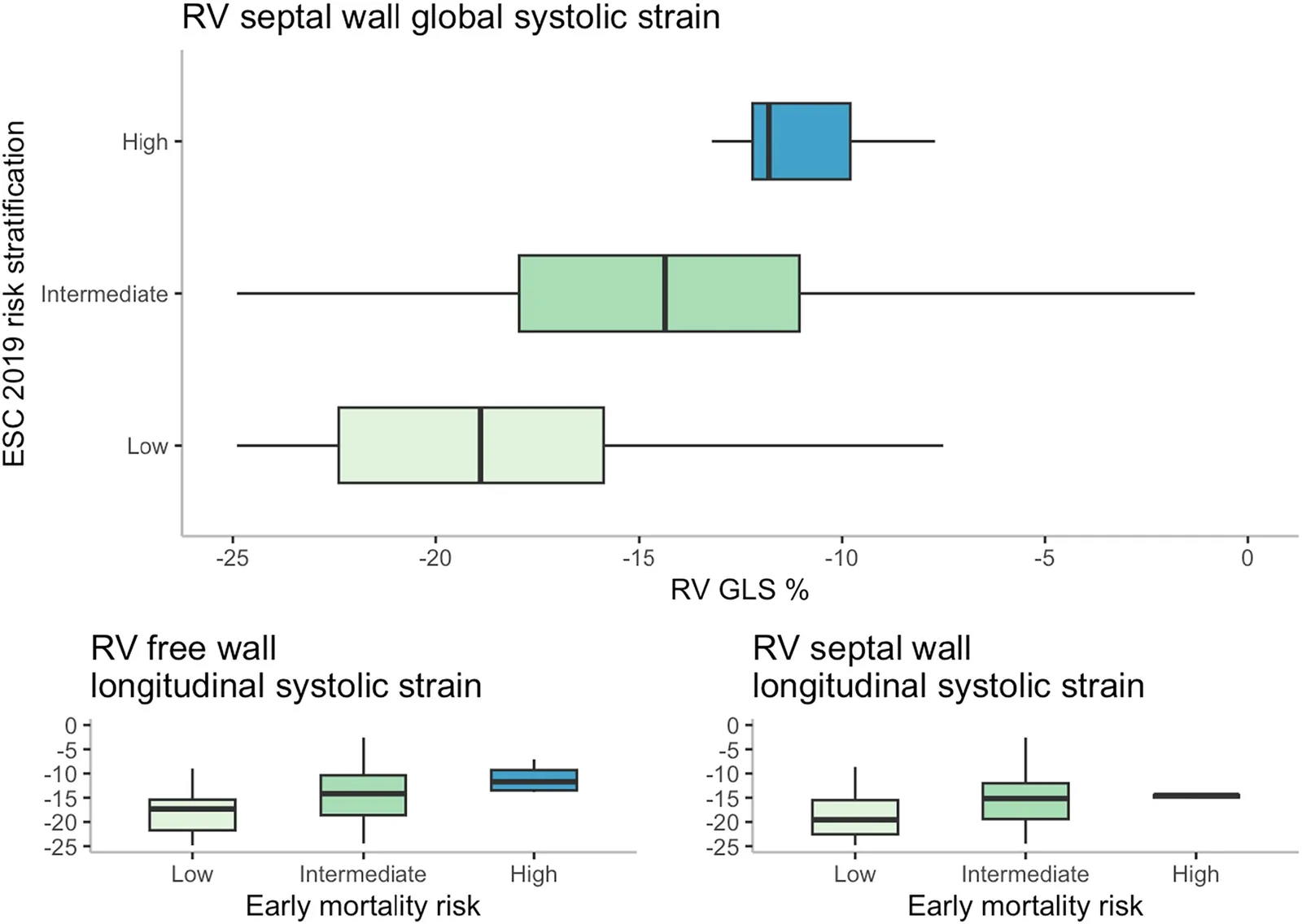
Difference in RV strain parameters at admission between patients according to the 2019 European Society of Cardiology Risk Stratification.

**Table 2:** Echocardiographic parameters of patients at follow-up

Characteristic	All patients			Non-PPEI/CTEPH			PPEI/CTEPH		
	Baseline, *N* = 144^a^	3 months, *N* = 141^a^	12 months, *N* = 130^a^	Baseline, *N* = 128^a^	3 months, *N* = 125^a^	12 months, *N* = 119^a^	Baseline, *N* = 16^a^	3 months, *N* = 16^a^	12 months, *N* = 11^a^
RA end-systolic area (cm^2^)	16.5 (14.1, 21.0)	14.6 (12.0, 17.0)	15.0 (12.1, 17.0)	16.0 (14.1, 20.4)	14.5 (12.0, 16.2)	14.9 (12.1, 17.0)	19.0 (13.8, 22.2)	16.3 (11.8, 20.1)	15.0 (13.3, 22.5)
RV-D1 end-diastolic diameter (mm)	40 (36, 44)	36 (32, 40)	36 (32, 40)	40 (36, 43)	36 (32, 39)	36 (32, 39)	41 (39, 44)	38 (36, 42)	35 (31, 42)
RV/LV end-diastolic ratio	0.90 (0.81, 1.00)	0.80 (0.72, 0.90)	0.80 (0.72, 0.89)	0.90 (0.81, 1.00)	0.80 (0.70, 0.90)	0.80 (0.72, 0.89)	0.88 (0.80, 1.13)	0.80 (0.74, 0.90)	0.83 (0.80, 0.90)
Paradoxical motion on echo	24/144 (17%)	5/138 (3.6%)	0/129 (0%)	22/128 (17%)	3/122 (2.5%)	0/118 (0%)	2/16 (12%)	2/16 (12%)	0/11 (0%)
TAPSE (mm)	20 (18, 23)	22 (20, 26)	22 (20, 25)	20 (18, 23)	23 (20, 26)	22 (20, 25)	20 (18, 26)	22 (18, 25)	23 (16, 26)
Pericardial effusion	5/143 (3.5%)	2/138 (1.4%)	1/129 (0.8%)	4/127 (3.1%)	1/122 (0.8%)	1/118 (0.8%)	1/16 (6.2%)	1/16 (6.2%)	0/11 (0%)
Tricuspid regurgitation velocity (m/s)	2.86 (2.50, 3.16)	2.53 (2.31, 2.74)	2.50 (2.31, 2.70)	2.86 (2.56, 3.14)	2.56 (2.32, 2.74)	2.50 (2.35, 2.70)	2.80 (2.43, 3.40)	2.43 (2.29, 2.63)	2.78 (2.30, 2.80)
RV free wall longitudinal systolic strain, basal segment (%)	−20 (−26, −14)	−20 (−26, −14)	−20 (−26, −15)	−20 (−26, −14)	−20 (−26, −15)	−21 (−27, −16)	−18 (−25, −15)	−16 (−21, −12)	−9 (−18, −3)
RV free wall longitudinal systolic strain, middle segment (%)	−16 (−21, −10)	−18 (−23, −13)	−18 (−21, −13)	−16 (−23, −10)	−18 (−23, −14)	−18 (−21, −13)	−13 (−17, −11)	−14 (−19, −11)	−6 (−13, −1)
RV free wall longitudinal systolic strain, apical segment (%)	−14 (−19, −8)	−18 (−23, −13)	−17 (−21, −11)	−14 (−20, −9)	−19 (−24, −13)	−17 (−21, −13)	−11 (−15, 7)	−14 (−17, −8)	−7 (−11, −1)
RV free wall longitudinal systolic strain (%)	−16 (−21, −11)	−18 (−23, −14)	−18 (−22, −15)	−17 (−23, −11)	−19 (−24, −15)	−18 (−22, −16)	−12.9 (−15.7, −9.4)	−14.1 (−17.6, −11.3)	−4.5 (−11.8, −3.4)
RV septal wall longitudinal systolic strain, basal segment (%)	−17 (−21, −11)	−16 (−21, −13)	−18 (−21, −13)	−17 (−21, −11)	−17 (−21, −14)	−18 (−21, −13)	−14 (−19, −11)	−13 (−15, −10)	−6 (−9, −2)
RV septal wall longitudinal systolic strain, middle segment (%)	−17.2 (−21.9, −12.3)	−18.7 (−21.9, −14.5)	−18.6 (−20.8, −14.8)	−18.0 (−22.0, −12.4)	−19.0 (−22.0, −15.3)	−18.7 (−20.9, −15.2)	−13 (−17, −10)	−13 (−16, −11)	−6 (−18, −1)
RV septal wall longitudinal systolic strain, apical segment (%)	−19 (−25, −14)	−21 (−27, −16)	−22 (−26, −16)	−19 (−26, −14)	−23 (−28, −17)	−22 (−26, −17)	−15 (−20, −11)	−14 (−16, −11)	−10 (−23, −8)
RV septal wall longitudinal systolic strain (%)	−18 (−23, −13)	−20 (−23, −15)	−19 (−22, −14)	−18.3 (−22.7, −13.7)	−20.0 (−22.7, −16.2)	−18.9 (−22.0, −15.5)	−14.9 (−18.1, −10.7)	−13.0 (−15.2, −11.2)	−6.7 (−14.2, −2.9)
RV global longitudinal systolic strain (%)	−16 (−21, −12)	−18 (−22, −14)	−18 (−21, −14)	−16.5 (−22.2, −12.0)	−18.9 (−23.1, −14.9)	−18.3 (−21.1, −14.4)	−12.9 (−16.9, −10.9)	−12.6 (−17.6, −10.1)	−6.6 (−10.9, −4.5)
RV global longitudinal systolic strain rate (s^−1^)	−1.80 (−2.30, −1.40)	−1.90 (−2.30, −1.60)	−2.00 (−2.53, −1.60)	−1.90 (−2.30, −1.40)	−1.90 (−2.30, −1.60)	−2.10 (−2.60, −1.70)	−1.55 (−1.80, −1.18)	−1.85 (−2.05, −1.63)	−1.10 (−1.70, −1.00)
RV end-diastolic volume (mL)	44 (31, 63)	38 (25, 52)	38 (25, 54)	43 (31, 62)	37 (25, 50)	38 (24, 53)	57 (35, 72)	67 (38, 85)	39 (28, 62)
RV end-systolic volume (mL)	25 (16, 41)	18 (11, 33)	18 (12, 33)	24 (15, 40)	17 (11, 30)	16 (11, 32)	33 (21, 48)	37 (25, 48)	29 (22, 39)
RV ejection fraction (%)	40 (28, 56)	50 (39, 60)	47 (32, 60)	41 (29, 56)	51 (40, 62)	51 (36, 60)	34 (27, 47)	41 (30, 50)	27 (13, 35)
RV free wall time to peak strain, basal segment (s)	0.36 (0.32, 0.43)	0.37 (0.33, 0.44)	0.37 (0.33, 0.43)	0.36 (0.31, 0.42)	0.37 (0.34, 0.44)	0.37 (0.33, 0.43)	0.38 (0.33, 0.50)	0.33 (0.31, 0.56)	0.38 (0.32, 0.45)
RV free wall time to peak strain, middle segment (s)	0.37 (0.31, 0.42)	0.38 (0.33, 0.43)	0.38 (0.32, 0.43)	0.37 (0.31, 0.42)	0.38 (0.33, 0.43)	0.38 (0.32, 0.43)	0.36 (0.33, 0.44)	0.36 (0.28, 0.43)	0.42 (0.32, 0.67)
RV free wall time to peak strain, apical segment (s)	0.35 (0.30, 0.43)	0.38 (0.32, 0.45)	0.38 (0.33, 0.46)	0.35 (0.29, 0.42)	0.38 (0.32, 0.44)	0.38 (0.33, 0.46)	0.38 (0.33, 0.49)	0.34 (0.23, 0.43)	0.45 (0.37, 0.56)
RV free wall time to peak strain (s)	0.37 (0.33, 0.42)	0.38 (0.34, 0.42)	0.38 (0.33, 0.44)	0.37 (0.33, 0.42)	0.38 (0.34, 0.42)	0.37 (0.33, 0.43)	0.39 (0.32, 0.48)	0.35 (0.32, 0.47)	0.41 (0.32, 0.60)
RV septal wall time to peak strain, basal segment (s)	0.37 (0.32, 0.45)	0.37 (0.33, 0.44)	0.37 (0.32, 0.46)	0.37 (0.32, 0.44)	0.38 (0.33, 0.44)	0.37 (0.32, 0.45)	0.41 (0.34, 0.46)	0.33 (0.31, 0.37)	0.46 (0.31, 0.66)
RV septal wall time to peak strain, middle segment (s)	0.34 (0.31, 0.39)	0.37 (0.33, 0.42)	0.36 (0.32, 0.43)	0.34 (0.30, 0.39)	0.37 (0.33, 0.42)	0.36 (0.32, 0.43)	0.41 (0.34, 0.46)	0.34 (0.32, 0.57)	0.40 (0.33, 0.44)
RV septal wall time to peak strain, apical segment (s)	0.34 (0.30, 0.40)	0.37 (0.34, 0.43)	0.37 (0.32, 0.42)	0.34 (0.30, 0.40)	0.38 (0.34, 0.42)	0.37 (0.32, 0.42)	0.35 (0.33, 0.46)	0.36 (0.34, 0.61)	0.41 (0.35, 0.44)
RV septal wall time to peak strain (s)	0.36 (0.31, 0.42)	0.38 (0.34, 0.42)	0.37 (0.33, 0.43)	0.36 (0.31, 0.41)	0.38 (0.34, 0.43)	0.37 (0.33, 0.43)	0.41 (0.34, 0.45)	0.35 (0.32, 0.38)	0.42 (0.33, 0.59)
RV peak systolic strain dyssynchrony index (%)	55 (29, 85)	38 (19, 65)	46 (18, 85)	52 (29, 80)	37 (19, 62)	46 (17, 83)	80 (46, 105)	40 (20, 86)	50 (29, 118)
Time to peak longitudinal systolic strain difference (s)	0.04 (0.02, 0.09)	0.03 (0.01, 0.06)	0.02 (0.01, 0.06)	0.04 (0.02, 0.09)	0.03 (0.01, 0.06)	0.02 (0.01, 0.05)	0.06 (0.02, 0.12)	0.05 (0.02, 0.09)	0.06 (0.03, 0.09)
LV global longitudinal systolic strain (%)	−17.2 (−20.2, −14.4)	−18.7 (−20.8, −15.6)	−18.4 (−21.4, −14.6)	−17.6 (−20.4, −15.2)	−19.0 (−21.1, −16.6)	−18.6 (−21.5, −15.4)	−12 (−16, −10)	−15 (−19, −11)	−7 (−14, −4)
TAPSE to sPAP (mm/mmHg)	0.59 (0.42, 0.78)	0.76 (0.60, 0.96)	0.77 (0.61, 0.93)	0.60 (0.42, 0.78)	0.75 (0.60, 0.94)	0.77 (0.61, 0.93)	0.58 (0.36, 0.93)	0.90 (0.70, 1.00)	0.82 (0.42, 0.94)
RVGLS to sPAP (%/mmHg)	0.44 (0.26, 0.69)	0.64 (0.40, 0.80)	0.63 (0.43, 0.76)	0.44 (0.27, 0.71)	0.65 (0.44, 0.83)	0.64 (0.47, 0.76)	0.40 (0.20, 0.54)	0.32 (0.20, 0.75)	0.18 (0.12, 0.41)
RVFWLS to sPAP (%/mmHg)	0.44 (0.24, 0.65)	0.63 (0.39, 0.82)	0.64 (0.46, 0.76)	0.44 (0.26, 0.66)	0.65 (0.44, 0.84)	0.66 (0.51, 0.77)	0.43 (0.18, 0.54)	0.29 (0.24, 0.81)	0.15 (0.09, 0.33)

^a^Median (IQR); *n*/*N* (%)

Numbers of missing values are reported in Supplementary data online, *Table S3*.

IVC, vena cava inferior, LAVI, left atrial volume index, LV, left ventricle, RA, right atrium, RV, right ventricle, RVFWLS, right ventricular free wall longitudinal systolic strain, RVGLS, right ventricular global longitudinal systolic strain, sPAP, systolic pulmonary arterial pressure, TAPSE, tricuspid annular plane systolic excursion.

Among the 144 patients with at least one follow-up assessment, RV function demonstrated a consistent improvement over time, particularly with respect to strain parameters, volumetric parameters, and ventriculoarterial coupling (*Table 2*). RV longitudinal deformation improved across follow-up, with RVGLS strain improving from −16% at baseline to −18% at 3 months and remaining stable at −18% at 12 months. Similar improvements were observed for RVFWLS (from −16 to −18%). Notably, the apical segment of the RV free wall exhibited a pronounced improvement, improving from −14% at baseline to −18% at 3 months and −17% at 12 months. In parallel, RVGLS rate modestly increased from −1.80 s^−1^ at baseline to −2.00 s^−1^ at 12 months.

RV volumetric remodelling accompanied these functional changes, with RV end-diastolic volume decreasing from 44 mL at baseline to 38 mL at 3 months and remaining stable at 12 months, while RV end-systolic volume declined from 25 to 18 mL. Consistent with these findings, RV ejection fraction improved from 40% at baseline to 50% at 3 months and was sustained at 47% at 12 months. Ventriculoarterial coupling parameters showed a marked improvement over time, with TAPSE/sPAP increasing from 0.59 to 0.77 mm/mmHg, RVGLS/sPAP from 0.44 to 0.63%/mmHg, and RVFWLS/sPAP from 0.44 to 0.64%/mmHg between baseline and 12 months.

Conventional echocardiographic markers of RV dysfunction demonstrated parallel changes, including reductions in RA and RV dimensions, a decrease in RV/LV end-diastolic ratio, a decline in tricuspid regurgitation velocity, and an improvement in TAPSE over follow-up.

### PPEI/CTEPH vs. non-PPEI/CTEPH

Of patients’ demographic characteristics, patients without PPEI/CTEPH were younger compared with those with PPEI/CTEPH (60 years vs. 69 years) and had a similar gender distribution (women: 40 vs. 38%). RV dysfunction at the initial PE event was also equally prevalent in both groups (57 vs. 69%). There were no statistically significant differences in comorbidities and PE risk factors between the groups, except that patients in the PPEI/CTEPH group were more likely to have a history of congestive heart failure or coronary heart disease (PPEI/CTEPH: 44%, non-PPEI/CTEPH: 7%).

In total, 16 patients developed PPEI (*n* = 15) or CTEPH (*n* = 3) during follow-up. In non-PPEI/CTEPH patients, strain parameters exhibited a trend towards augmentation (*Table 3*), especially the RVFWLS with a monthly increase of −0.57% (95% CI −1.20 to 0.06, *P* = 0.076). RVGLS rate also improved modestly (−0.06 s^−1^ per month, 95% CI −0.11 to −0.01, *P* = 0.019). In contrast, PPEI/CTEPH patients exhibited a progressive deterioration in RV deformation, with RVGLS increasing (worsening) by 1.32% per month (95% CI −0.04 to 2.67, *P* = 0.056), reflecting a near-opposite trajectory compared with non-PPEI/CTEPH patients (*Figure 3*).

**Figure 3:**
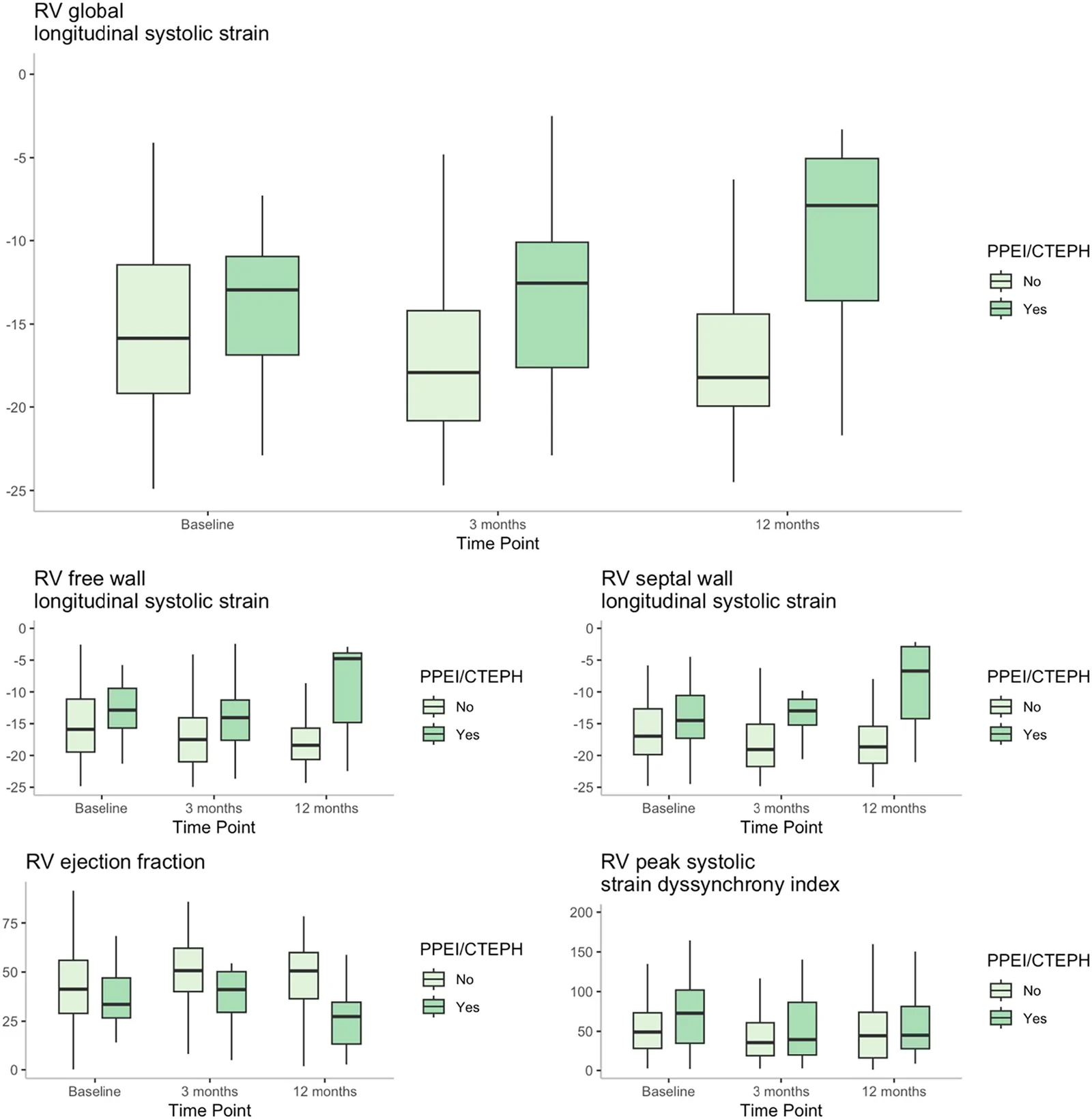
Difference in RV strain parameters at admission and follow-up between patients with and without PPEI/CTEPH. CTEPH, chronic thromboembolic pulmonary hypertension; PPEI, post-pulmonary embolism impairment; RV, right ventricle.

**Table 3:** Change of strain analysis parameters across visits

Parameter	Non-PPEI/CTEPH estimate (95% CI)	*P*	PPEI/CTEPH estimate (95% CI)	*P*
RV end-diastolic diameter (mm)	**−1.55** (**−1.99 to −1.10)**	**<0**.**001**	−0.67 (−2.30 to 0.96)	0.408
TAPSE (mm)	**0.79 (0.44 to 1.13)**	**<0**.**001**	−0.26 (−1.56 to 1.03)	0.684
Tricuspid regurgitation velocity (m/s)	**−0.11 (−0.14 to −0.08)**	**<0**.**001**	−0.05 (−0.16 to 0.06)	0.330
RV free wall longitudinal systolic strain	−0.57 (−1.20 to 0.06)	0.076	0.93 (−0.79 to 2.66)	0.277
RV septal wall longitudinal systolic strain	−0.17 (−0.68 to 0.34)	0.511	1.34 (−0.05 to 2.74)	0.059
RV global longitudinal systolic strain	−0.32 (−0.89 to 0.25)	0.270	1.32 (−0.04 to 2.67)	0.056
RV global longitudinal systolic strain rate	**−0.06 (−0.11 to −0.01)**	**0**.**019**	−0.04 (−0.15 to 0.06)	0.407
RV end-diastolic volume	**−2.76 (−4.39 to −1.13)**	**0**.**001**	−0.30 (−6.94 to 6.34)	0.927
RV end-systolic volume	**−2.67 (−4.04 to −1.29)**	**<0**.**001**	−0.36 (−4.75 to 4.03)	0.868
RV ejection fraction	**2.16 (0.65 to 3.67)**	**0**.**005**	−2.15 (−6.44 to 2.14)	0.315
RV free wall time to peak strain	0.01 (−0.00 to 0.02)	0.178	0.03 (−0.03 to 0.09)	0.319
RV septal wall time to peak strain	0.01 (−0.01 to 0.02)	0.231	0.03 (−0.03 to 0.09)	0.360
RV peak systolic strain dyssynchrony index	−3.84 (−8.42 to 0.75)	0.101	−2.36 (−29.25 to 24.52)	0.859
Time to peak longitudinal systolic strain difference	−0.01 (−0.01 to 0.00)	0.067	−0.01 (−0.02 to 0.01)	0.479
TAPSE/sPAP	**0.06 (0.03 to 0.08)**	**<0**.**001**	0.03 (−0.02 to 0.08)	0.263
RVGLS/sPAP	**0.05 (0.03 to 0.08)**	**<0**.**001**	−0.02 (−0.09 to 0.05)	0.501
RVFWLS/sPAP	**0.06 (0.03 to 0.09)**	**<0**.**001**	−0.01 (−0.09 to 0.06)	0.711

RV, right ventricle, RVFWLS, right ventricular free wall longitudinal systolic strain, RVGLS, right ventricular global longitudinal systolic strain, sPAP, systolic pulmonary arterial pressure, TAPSE, tricuspid annular plane systolic excursion. Values in bold indicate significance.

Volumetric parameters mirrored these functional changes. In non-PPEI/CTEPH patients, RV end-diastolic and end-systolic volumes declined over time (−2.76 mL/month, 95% CI −4.39 to −1.13, *P* = 0.001; −2.67 mL/month, 95% CI −4.04 to −1.29, *P* < 0.001, respectively), with a corresponding improvement in RV ejection fraction (+2.16%, 95% CI 0.65–3.67, *P* = 0.005). In PPEI/CTEPH patients, volumetric parameters remained largely unchanged, and RV ejection fraction showed a non-significant decrease (−2.15%, 95% CI −6.44 to 2.14, *P* = 0.315).

Regarding ventriculoarterial coupling, non-PPEI/CTEPH patients demonstrated improvement across all parameters: TAPSE/sPAP increased by 0.06 mm/mmHg/month (95% CI 0.03–0.08, *P* < 0.001), RVGLS/sPAP by 0.05%/mmHg/month (95% CI 0.03–0.08, *P* < 0.001), and RVFWLS/sPAP by 0.06%/mmHg/month (95% CI 0.03–0.09, *P* < 0.001). In contrast, PPEI/CTEPH patients exhibited, on average, a decline in strain-based ventriculoarterial coupling parameters (RVGLS/sPAP −0.02%/mmHg/month, 95% CI −0.09 to 0.05, *P* = 0.501; RVFWLS/sPAP −0.01%/mmHg/month, 95% CI −0.09 to 0.06, *P* = 0.711), whereas TAPSE/sPAP showed a slight positive trend (+0.03 mm/mmHg/month, 95% CI −0.02 to 0.08, *P* = 0.263), highlighting that strain-based metrics were more sensitive to the progressive RV–PA uncoupling in PPEI/CTEPH (*Figure 4*).

**Figure 4:**
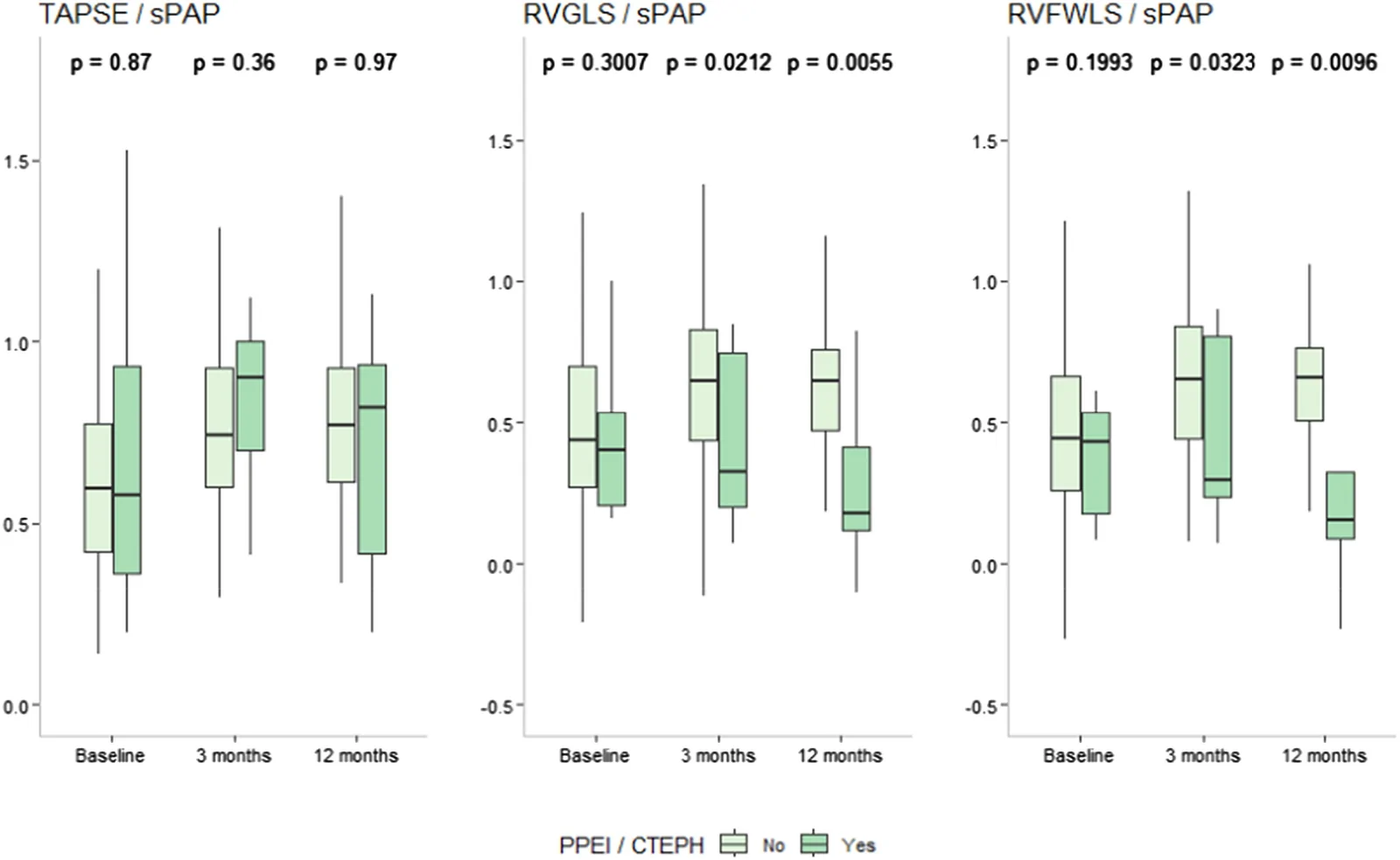
Difference in ventriculoarterial coupling parameters as assessed by echocardiography at admission and follow-up between patients with and without PPEI/CTEPH. CTEPH, chronic thromboembolic pulmonary hypertension; PPEI, post-pulmonary embolism impairment; RVFWLS, right ventricular free wall longitudinal systolic strain; RVGLS, right ventricular global longitudinal systolic strain; sPAP, systolic pulmonary arterial pressure; TAPSE, tricuspid annular plane systolic excursion.

In line with the above, RVGLS at 3 months was significantly associated with the combined CTEPH/PPEI endpoint over the 2-year follow-up period (OR 1.10, 95% CI 1.01–1.21, *P* = 0.034) in the multivariable model. Notably, strain-derived ventriculoarterial coupling indices at 3 months, including RVGLS/sPAP and RVFWLS/sPAP, also showed a strong trend towards the development of the CTEPH/PPEI endpoint (OR 0.11, 95% CI 0.01–0.91; *P* = 0.053; OR 0.16, 95% CI 0.02–1.17, *P* = 0.086, respectively). In contrast, TAPSE/sPAP showed no significant association (OR 0.72, 95% CI 0.06–4.66, *P* = 0.76). However, RVGLS did not correlate significantly with other outcomes, such as quality of life or rehospitalization (65/144 patients, 45%). Analysis of the endpoint ‘death’ was not feasible due to the occurrence of only one event.

## Discussion

In this echocardiographic sub-study of the FOCUS cohort of acute PE survivors, several important observations could be made. First, RV deformation assessed by speckle-tracking echocardiography was closely related to the severity of the index event, showing a clear and graded deterioration across early mortality risk categories and significant correlations with established biomarkers of adverse prognosis. Secondly, among patients with available follow-up, RV function demonstrated a robust and largely sustained recovery over time, characterized by improvement in strain parameters, volumetric remodelling, ejection fraction, and ventriculoarterial coupling. Thirdly, and most importantly, patients in whom PPEI or CTEPH was confirmed during long-term follow-up displayed a fundamentally different trajectory, with progressive worsening of RV strain and strain-based ventriculoarterial coupling despite relatively preserved or even slightly improving TAPSE-based indices. Collectively, these findings, although limited by the small number of PPEI/CTEPH events, underscore a potential sensitivity of RV strain, particularly when this is integrated into ventriculoarterial coupling metrics. Our results suggest that RV strain could be further studied as an early marker of maladaptive RV remodelling and impaired RV–pulmonary arterial interaction after acute PE.

Traditional echocardiographic RV parameters, including TAPSE, RV/LV ratio, and RV size, have established prognostic value in acute PE, with study- and patient-level meta-analyses displaying the association of RV dysfunction with adverse outcomes,^19,20^ while a combination of at least two parameters has been shown to identify those at increased risk of early death.^20^ In our study, strain parameters were markedly impaired at baseline and demonstrated a clear, stepwise deterioration across early mortality risk categories, as well as strong correlation with prognostic biomarkers, supporting their close association with acute RV pressure overload and myocardial stress. The value of assessing strain parameters to better stratify acute PE and identify patients at risk of adverse outcome has been shown for both intermediate risk PE,^21^ as well as high risk PE.^22^

In the present cohort, standard RV parameters demonstrated significant improvement during follow-up in the overall population. These findings are consistent with the established concept that acute RV pressure overload is, in most patients, a reversible phenomenon following adequate reperfusion and unloading of the pulmonary circulation^23^; for example, in a cohort of patients with intermediate-risk PE, echocardiographic parameters of RV function normalized within 6 days in most patients; however, some patients continued to display RV dysfunction over the long-term.^24^ In other studies, abnormalities in pre-discharge echocardiography persisted in almost 40% of patients,^25^ and impaired echocardiographic parameters at discharge were associated with higher likelihood of exercise limitation at 6 months.^26^

In our study, while general RV parameters showed recovery overall, their ability to early discriminate patients who developed PPEI or CTEPH in the 2-year follow-up appeared limited. In particular, TAPSE-derived ventriculoarterial coupling (TAPSE/sPAP) showed improvement over time even in patients who ultimately developed PPEI/CTEPH, while the strain-derived one declined over time in PPEI/CTEPH patients. This discordance highlights that strain-based coupling metrics may detect subtle but clinically relevant RV–PA uncoupling that is not captured by TAPSE-based measures. In addition, a strong association was observed between RVGLS measured at the early follow-up time point of 3 months and the subsequent development of the PPEI/CTEPH endpoint, with similar associations noted for strain-derived ventriculoarterial coupling parameters. In contrast, TAPSE/sPAP showed no meaningful association with those late PE-related outcomes. Of note, in the FOCUS cohort, the PPEI endpoint was defined based on documentation at two successive follow-up assessments, and therefore, most patients reached the endpoint at least 1 year after the index PE event. As such, speckle-tracking echocardiographic analysis may represent a particularly valuable tool allowing early identification of patients in whom adverse RV remodelling and progressive ventriculoarterial uncoupling persist beyond the acute phase and precede the clinical manifestation of late complications, such as PPEI or CTEPH. The above also reflects the established prognostic value of RV strain in overt PH^27^ and may spark the hypothesis that strain parameters could be a potentially valuable tool for prognostically assessing patients with cardiopulmonary limitations after PE without overt PH.^6^

There is a growing interest for developing post-PE follow-up algorithms. The 2019 ESC guidelines proposed for the first time an expert consensus derived algorithm, which places echocardiography in the centre of evaluations after acute PE, and is designed to assess patients with functional limitations newly or persisting after the index event.^2^ In a recent study, using data from the FOCUS cohort, we showed that this approach primarily identifies patients with more severe post-PE sequelae, while a substantial proportion of patients with milder but clinically relevant impairment, including some who later developed CTEPH, may remain undetected.^28^ Although some approaches using ECG and BNP, optionally refined by baseline computed-tomography pulmonary angiogram, may help better select patients eligible for echocardiography, they were primarily designed to exclude CTEPH and may also miss patients with functional impairment without overt pulmonary hypertension.^29,30^ Such patients could benefit from additional testing, particularly cardiopulmonary exercise testing (CPET). The SEARCH algorithm (currently under investigation) places CPET at the centre and aims not only to identify patients with overt CTEPH at the severe end of the spectrum but also those with functional limitations and incomplete physiologic recovery, corresponding to phenotypes of chronic thromboembolic disease.^31^ In this context, the prospective evaluation and incorporation of RV strain, alongside more advanced echocardiographic techniques such as three-dimensional imaging and stress echocardiography, represent a promising avenue for improving post-PE follow-up, as these approaches may provide greater sensitivity and prognostic value compared with conventional echocardiography.^32^

This analysis has several limitations. FOCUS was a prospective cohort study on patients with PE, and the absence of a control group without PE prevents us from determining an independent association between PE and RVGLS. Instead, our findings aim to estimate the temporal changes in strain specific to this patient population. The limited number of PPEI/CTEPH patients limits the power of the analysis to detect differences among the two groups of patients and discover associations with quality of life and other outcomes. Further studies, potentially with larger event numbers, are needed to validate our findings. Additionally, selection bias cannot be ruled out, as not all patients had adequate echocardiographic data at all visits, leading to the exclusion of ∼35% of patients due to missing or poor-quality imaging. Also, missing measurements at individual follow-up visits were not imputed. Furthermore, all images analysed in this sub-study were obtained from the Mainz centre only, which may introduce centre-specific variability and limit the generalizability of the findings. Finally, although speckle-tracking echocardiography provides sensitive measures of RV function, inter- and intra-observer variability and differences in vendor software may affect reproducibility, and these results may not be directly transferrable to other platforms or clinical settings.^33^

In conclusion, our findings highlight the potential of RV strain, particularly when integrated with ventriculoarterial coupling parameters, as a sensitive marker of early maladaptive RV remodelling following acute PE. Strain-based metrics may complement conventional echocardiographic parameters in order to enhance the identification of patients at risk for long-term complications such as PPEI and CTEPH. Incorporating RV strain, alongside advanced echocardiographic techniques such as three-dimensional imaging and stress echocardiography, into post-PE follow-up strategies should be further studied in order to enhance risk stratification, enable timely interventions, and ultimately improve long-term outcomes for PE survivors.

## Supplementary Material

qyag166_Supplementary_Data

## Data Availability

The data underlying this article may be available upon available request to the corresponding authors.

## Lead author biography

Lukas Hobohm is an interventional cardiologist and clinical researcher affiliated with the University Medical Center Mainz and the Center for Thrombosis and Hemostasis in Mainz, Germany. The scientific focus of Dr Hobohm is on optimizing risk stratification and management strategies for patients with acute PE. In this context Dr Hobohm also focusing on epidemiological as well as translational ‘bench-to-bedside’ research of thrombotic and thromboembolic disorders. Furthermore Dr Hobohm is working as a senior physician at the Department of Cardiology at the Medical University in Mainz, is co-leading the interventional PE-programme, and is co-heading as a clinical investigator ongoing prospective cohort studies and interventional trials at the Center for Thrombosis and Hemostasis (CTH) in Mainz.
