# Supplementary material for: Right ventricular strain analysis in acute pulmonary embolism and its role for late complications
Source: Eur Heart J Imaging Methods Pract. 2026 Sep 22;4(4):qyag166. doi: 10.1093/ehjimp/qyag166 (PMC13673565; doi:10.1093/ehjimp/qyag166)
Supplement: Supplementary file 1 [file qyag166_supplementary_data.docx]

**Supplemental Material of “Right ventricular global longitudinal strain analysis in acute pulmonary embolism and its role for late complications”**

**Supplemental Table 1. Baseline characteristics and differences of included and excluded patients.**

| **Characteristic** | Excluded N = 97*^1^* | Included N = 175*^1^* | **p-value***^2^* |
| --- | --- | --- | --- |
| Age (years) | 65 (53, 74) | 61 (50, 73) | 0.3 |
| Women | 36/97 (37%) | 71/175 (41%) |  |
| BMI (kg/m²) | 27 (25, 32) | 28 (26, 33) | 0.13 |
| Systolic blood pressure (mmHg) | 132 (118, 160) | 138 (124, 155) | 0.3 |
| Diastolic blood pressure (mmHg) | 77 (68, 87) | 81 (73, 91) | 0.010 |
| SaO2 at room air (%) | 96.0 (94.0, 98.0) | 96.0 (93.0, 97.0) | 0.3 |
| Either Troponin T or Troponin I elevation | 41/90 (46%) | 81/167 (49%) | 0.7 |
| Right ventricular dysfunction on imaging | 41/97 (42%) | 99/175 (57%) | 0.024 |
| sPESI (non-missing if at least one component non-missing) |  |  | 0.082 |
| 0 | 40/97 (41%) | 97/175 (55%) |  |
| 1 | 39/97 (40%) | 55/175 (31%) |  |
| 2 | 13/97 (13%) | 18/175 (10%) |  |
| 3 | 5/97 (5.2%) | 3/175 (1.7%) |  |
| 4 | 0/97 (0%) | 2/175 (1.1%) |  |
| PE early mortality risk |  |  | 0.066 |
| Low | 14/97 (14%) | 41/175 (23%) |  |
| Intermediate | 76/97 (78%) | 129/175 (74%) |  |
| High | 7/97 (7.2%) | 5/175 (2.9%) |  |
| Cancer | 16/97 (16%) | 12/175 (6.9%) | 0.012 |
| Oestrogen-containing medication | 7/97 (7.2%) | 17/175 (9.7%) | 0.5 |
| History of pregnancy or lactation | 0/97 (0%) | 2/175 (1.1%) | 0.5 |
| History of long-distance travel | 13/97 (13%) | 21/175 (12%) | 0.7 |
| Major trauma or major surgery | 19/97 (20%) | 28/175 (16%) | 0.5 |
| History of immobilization | 30/97 (31%) | 54/175 (31%) | >0.9 |
| History of VTE | 28/96 (29%) | 56/175 (32%) | 0.6 |
| Chronic pulmonary disease | 13/97 (13%) | 22/175 (13%) | 0.8 |
| History of CHF or CHD | 15/97 (15%) | 18/174 (10%) | 0.2 |
| Arterial hypertension | 58/97 (60%) | 109/175 (62%) | 0.7 |
| Diabetes mellitus | 13/97 (13%) | 30/175 (17%) | 0.4 |
| Chronic liver disease | 7/97 (7.2%) | 9/175 (5.1%) | 0.5 |
| History of CKD or eGFR < 50 at baseline | 19/97 (20%) | 32/175 (18%) | 0.8 |
| Chronic inflammatory disease | 13/97 (13%) | 19/175 (11%) | 0.5 |
| Smoker | 16/97 (16%) | 32/175 (18%) | 0.7 |
| *^1^* Median (Q1, Q3); n/N (%) | | | |
| *^2^* Wilcoxon rank sum test; Pearson’s Chi-squared test; Fisher’s exact test | | | |

**Supplemental Table 2. Echocardiographic parameters of all patients at baseline.**

| **Characteristic** | **N = 175***^1^* |
| --- | --- |
| RA end-systolic area (cm^2^) | 17.0 (14.0, 20.2) |
| RV-D1 end-diastolic diameter (mm) | 40 (35, 43) |
| Thickness of RV free wall (mm) | 7.00 (5.00, 8.00) |
| RV/LV end-diastolic ratio | 0.90 (0.80, 1.00) |
| Paradoxical motion | 28/175 (16%) |
| TAPSE (mm) | 21 (18, 23) |
| Tricuspid regurgitation velocity (m/s) | 2.80 (2.50, 3.15) |
| Pericardial effusion | 7/173 (4.0%) |
| RV free wall longitudinal systolic strain, basal segment | -20 (-26, -14) |
| RV free wall longitudinal systolic strain, middle segment | -16 (-21, -11) |
| RV free wall longitudinal systolic strain, apical segment | -14 (-20, -8) |
| RV free wall longitudinal systolic strain | -16 (-21, -11) |
| RV septal wall longitudinal systolic strain, basal segment | -17 (-21, -11) |
| RV septal wall longitudinal systolic strain, middle segment | -17 (-22, -12) |
| RV septal wall longitudinal systolic strain, apical segment | -19 (-25, -14) |
| RV septal wall longitudinal systolic strain | -17.6 (-22.2, -13.6) |
| RV global longitudinal systolic strain | -16 (-21, -12) |
| RV global longitudinal systolic strain rate | -1.80 (-2.30, -1.40) |
| RV end-diastolic volume | 45 (31, 64) |
| RV end-systolic volume | 25 (15, 43) |
| RV ejection fraction | 41 (28, 57) |
| RV free wall time to peak strain, basal segment | 0.36 (0.31, 0.43) |
| RV free wall time to peak strain, middle segment | 0.37 (0.31, 0.42) |
| RV free wall time to peak strain, apical segment | 0.35 (0.29, 0.43) |
| RV free wall time to peak strain | 0.37 (0.32, 0.42) |
| RV septal wall time to peak strain, basal segment | 0.37 (0.32, 0.45) |
| RV septal wall time to peak strain, middle segment | 0.34 (0.30, 0.40) |
| RV septal wall time to peak strain, apical segment | 0.35 (0.29, 0.41) |
| RV septal wall time to peak strain | 0.36 (0.31, 0.42) |
| RV peak systolic strain dyssynchrony index | 54 (28, 85) |
| Time to peak longitudinal systolic strain difference | 0.04 (0.02, 0.09) |
| LV global longitudinal systolic strain | -17.1 (-20.3, -14.2) |
| Left atrial volume index | 29 (18, 41) |
| TAPSE / sPAP (mm/mmHg) | 0.59 (0.42, 0.76) |
| RVGLS / sPAP (%/mmHg) | 0.42 (0.27, 0.65) |
| RVFWLS / sPAP(%/mmHg) | 0.42 (0.25, 0.64) |
| *^1^* Median (IQR); n/N (%)  IVC = Vena cava inferior, LAVI = left atrial volume index, LV = left ventricle, RA = right atrium, RV = right ventricle, RVFWLS = right ventricular free wall longitudinal systolic strain, RVGLS = right ventricular global longitudinal systolic strain, sPAP = systolic pulmonary arterial pressure, TAPSE = tricuspid annular plane systolic excursion. | |

**Supplemental Table 3. Missingness of echocardiographic parameters at baseline and follow-up.**

| Echocardiographic parameter | Baseline N = 144*^1^* | 3 months N = 141*^1^* | 12 months N = 130*^1^* |
| --- | --- | --- | --- |
| RA end-systolic area (cm²) | 55 | 34 | 25 |
| RV-D1 end-diastolic diameter (mm) | 25 | 9 | 13 |
| RV/LV end-diastolic ratio | 47 | 20 | 22 |
| Paradoxical motion on echo (original) | 0 | 3 | 1 |
| TAPSE (mm) | 24 | 10 | 7 |
| Pericardial effusion | 1 | 3 | 1 |
| Tricuspid regurgitation velocity (m/s) | 39 | 19 | 20 |
| RV free wall longitudinal systolic strain, basal segment | 0 | 23 | 37 |
| RV free wall longitudinal systolic strain, middle segment | 0 | 23 | 38 |
| RV free wall longitudinal systolic strain, apical segment | 0 | 23 | 39 |
| RV free wall longitudinal systolic strain | 0 | 23 | 39 |
| RV septal wall longitudinal systolic strain, basal segment | 0 | 23 | 36 |
| RV septal wall longitudinal systolic strain, middle segment | 0 | 23 | 36 |
| RV septal wall longitudinal systolic strain, apical segment | 0 | 23 | 36 |
| RV septal wall longitudinal systolic strain | 0 | 23 | 36 |
| RV global longitudinal systolic strain | 0 | 23 | 39 |
| RV global longitudinal systolic strain rate | 0 | 23 | 38 |
| RV end-diastolic volume | 4 | 25 | 38 |
| RV end-systolic volume | 4 | 25 | 38 |
| RV ejection fraction | 4 | 25 | 38 |
| RV free wall time to peak strain, basal segment | 1 | 23 | 38 |
| RV free wall time to peak strain, middle segment | 1 | 23 | 38 |
| RV free wall time to peak strain, apical segment | 1 | 23 | 39 |
| RV free wall time to peak strain | 1 | 23 | 39 |
| RV septal wall time to peak strain, basal segment | 1 | 23 | 37 |
| RV septal wall time to peak strain, middle segment | 1 | 23 | 37 |
| RV septal wall time to peak strain, apical segment | 1 | 23 | 37 |
| RV septal wall time to peak strain | 1 | 23 | 37 |
| RV peak systolic strain dyssynchrony index | 1 | 23 | 37 |
| Time to peak longitudinal systolic strain difference | 13 | 34 | 52 |
| LV global longitudinal systolic strain | 4 | 18 | 35 |
| TAPSE / sPAP | 40 | 24 | 24 |
| RVGLS / sPAP | 23 | 38 | 49 |
| RVfwLS / sPAP | 23 | 38 | 49 |
